# Influence of estrogen-related receptor γ (*ESRRG*) rs1890552 A > G polymorphism on changes in fasting glucose and arterial stiffness

**DOI:** 10.1038/s41598-017-10192-6

**Published:** 2017-08-29

**Authors:** Minjoo Kim, Hye Jin Yoo, Minkyung Kim, Haengok Seo, Jey Sook Chae, Sang-Hyun Lee, Jong Ho Lee

**Affiliations:** 1 Research Center for Silver Science, Institute of Symbiotic Life-TECH, Yonsei University, Seoul, 03722 Korea; 2 National Leading Research Laboratory of Clinical Nutrigenetics/Nutrigenomics, Department of Food and Nutrition, College of Human Ecology, Yonsei University, Seoul, 03722 Korea; 3 Department of Food & Nutrition, Brain Korea 21 PLUS Project, College of Human Ecology, Yonsei University, Seoul, 03722 Korea; 4 Department of Family Practice, National Health Insurance Corporation, Ilsan Hospital, Goyang, 10444 Korea

## Abstract

To determine the effects of the estrogen-related receptor γ (*ESRRG*) rs1890552 A > G polymorphism on dietary advice-mediated changes in fasting glucose and arterial stiffness, 374 subjects with normal fasting glucose (NFG; control group, no treatment) and 142 subjects with impaired fasting glucose (IFG group, dietary advice) were followed for 3.5 years. At follow-up, the GG subjects in the IFG group showed a significant reduction in fasting glucose, which was greater than in the AA subjects. A significant association was observed between *ESRRG* rs1890552 A > G polymorphism and changes in fasting glucose, brachial-ankle pulse wave velocity (ba-PWV), and 8-epi-prostaglandin F_2α_ in the IFG subjects. At baseline, the GG subjects showed a higher ba-PWV than the AA subjects in the IFG group. At the 3.5-year follow-up, subjects with AA or AG showed significant increases in ba-PWV, whereas subjects with GG showed a decrease from baseline. This study suggests that the *ESRRG* rs1890552 A > G polymorphism may modulate interindividual differences in atrial stiffness, with a reduction in fasting glucose in response to dietary advice in subjects with IFG after a 3.5-year follow-up.

## Introduction

Estrogen-related receptor γ (ESRRG) is a member of the orphan nuclear hormone receptor family of steroid hormone receptors, which function as constitutive activators of transcription^1^ and play various roles in regulating homeostatic and metabolic processes^2, 3^. *ESRRG* has been suggested as a novel candidate gene for type 2 diabetes (T2D) based on a genome-wide association study (GWAS)^4, 5^. A recent cross-sectional study showed that the *ESRRG* rs1890552 A > G SNP was a novel candidate variant for impaired fasting glucose (IFG) and T2D, although this SNP was not equivalent to the SNPs found in the GWAS^4^. Increased fasting plasma glucose, which includes IFG, impaired glucose tolerance (IGT), and T2D, is a risk factor for arterial stiffness and cardiovascular disease (CVD)^6–9^. Additionally, fasting glucose was reported to have an independent positive association with arterial stiffness measured using the brachial-ankle pulse wave velocity (ba-PWV) in non-diabetic subjects after correcting for confounding variables, including age, gender, body mass index (BMI), blood pressure (BP), resting heart rate, high-sensitivity C-reactive protein (hs-CRP), lipid profile, and behavioral habits^10^.

The glucose status of patients with IFG or newly diagnosed T2D is likely affected by acute dietary changes, whereas hyperglycemia-related vascular changes, including arterial stiffness, reflect long-term dietary intake. However, little is known about the long-term effects of dietary advice-induced modulation of arterial stiffness. Arterial stiffness can be easily and noninvasively assessed by measuring PWV^11, 12^, and ba-PWV measurement is a suitable means of screening for vascular dysfunction and the development of atherosclerosis in a preventative setting^10, 13, 14^. According to the 2014 Korean National Health and Nutrition Examination Survey (KNHANES VI-2), carbohydrate-derived calories account for 63.8% of the total caloric intake of middle-aged Korean adults, and cooked refined rice is the main source of carbohydrates. Due to this high carbohydrate intake, the replacement of refined rice with whole grains and legumes has been suggested to reduce T2D risk factors^15^. Humans differ in their responses to diet, and many of these differences may be due to genetic polymorphisms^16, 17^. In our previous report, the *ESRRG* rs1890552 A > G SNP was strongly associated with 8-epi-prostaglandin F_2α_ (8-epi-PGF_2α_), a reliable marker of glycemic control and oxidation status in patients with T2D^18^ and of IGT^19^, and was considered a novel candidate variant for IFG and T2D^20^. Therefore, we investigated the influence of the *ESRRG* rs1890552 A > G polymorphism on changes in fasting glucose and arterial stiffness in response to dietary advice in subjects with IFG over a 3.5-year period.

## Methods

### Study population

In this study, 1,933 participants with normal fasting glucose (NFG), IFG, and newly diagnosed T2D between the ages of 30 and 69 years were recruited from the National Health Insurance Corporation Ilsan Hospital in Goyang, Korea, as previously described^20^. Hyperglycemia was defined, according to the American Diabetes Association^21^, as fasting glucose ≥126 mg/dL and IFG as fasting glucose between 100 and 125 mg/dL. A total of 374 subjects with NFG (control group, no treatment), 142 subjects with IFG (IFG group), and 13 subjects with T2D were followed for 3.5 years. To observe the sole effect of the *ESRRG* rs1890552 A > G polymorphism on IFG, 13 subjects with T2D were excluded from this study. Subjects with a diagnosis or history of CVD, liver disease, renal disease, pancreatitis, cancer, pregnancy or lactation, or regular use of any medication were excluded. Written informed consent was obtained from each participant included in the study, and the Institutional Review Board of Yonsei University and the National Health Insurance Corporation Ilsan Hospital approved the study protocol, which complied with the Declaration of Helsinki.

### Dietary advice and assessment of dietary intake and physical activity level

The subjects’ usual diets were assessed at baseline and follow-up using a semi-quantitative food frequency and 24-h recall method. At baseline, all subjects were given written and verbal instructions by a dietitian on how to complete a 3-day (2 weekdays and 1 weekend day) dietary record. Individualized and nutritionally balanced diets were planned for each subject at the initial visit. The dietary advice for the IFG group consisted of replacing refined rice with whole grains and legumes three times per day as a carbohydrate source and an increase in vegetable intake to at least 6 units (30–70 g/unit) per day to ensure a sufficient dietary fiber intake. The subjects were told to drink no more than one alcoholic beverage (15 g of alcohol) and to participate in a physical activity consisting of a regular 30-min walk after dinner each day. The energy values and nutrient contents of the dietary intake were calculated using the Computer-Aided Nutritional Analysis Program (CAN-pro 3.0, Korean Nutrition Society, Seoul, Korea). Total energy expenditure was calculated based on activity patterns, including the basal metabolic rate (by the Harris-Benedict equation), physical activity for 24 h, and the specific dynamic actions of food.

### Anthropometric measurements

Body weight (UM0703581; Tanita, Tokyo, Japan) and height (GL-150; G-tech International, Uijeongbu, Korea) were measured in the morning without clothing and shoes. BMI was calculated as body weight (kg) divided by height (m^2^). BP was measured twice using an automatic BP monitor (FT-200S; Jawon Medical, Gyeongsan, Korea) after a resting period of at least 20 min.

### Blood and urine collection

Venous blood samples were collected in EDTA-treated tubes and tubes without an anticoagulant after at least 12 h of overnight fasting. The samples were centrifuged to produce plasma and serum within 3 h after blood collection. Blood sample aliquots were stored at −70 °C prior to analysis. Urine samples were collected in polyethylene bottles that contained 1% butylated hydroxytoluene after an overnight fast of at least 12 h. The bottles were immediately covered with aluminum foil and stored at −20 °C until analysis.

### Clinical laboratory parameters

Serum triglycerides (Pureauto S TG-N kits; Daiichi, Tokyo, Japan) and total cholesterol (Pureauto S CHO-N kits; Daiichi, Tokyo, Japan) were analyzed via an enzymatic assay. Serum high-density lipoprotein (HDL) cholesterol levels (Cholestest N-HDL kits; Daiichi, Tokyo, Japan) were measured using selective inhibition; the resulting color reaction was monitored using a Hitachi 7600 Autoanalyzer (Hitachi Ltd., Tokyo, Japan). Low-density lipoprotein (LDL) cholesterol levels were calculated using the Friedewald formula, where LDL cholesterol = total cholesterol − [HDL cholesterol + (triacylglycerol/5)]. Serum glucose and insulin levels were measured using commercial kits (Siemens, Tarrytown, NY, USA; DIAsource ImmunoAssays S.A., Louvainla-Neuve, Belgium), and the resulting color reaction was monitored using a Hitachi 7600 Autoanalyzer (Hitachi Ltd., Tokyo, Japan) for glucose and an SR-300 system (Stratec, Birkenfeld, Germany) for insulin. Homeostasis model assessment (HOMA) was used to assess insulin resistance via the equation HOMA-IR = [fasting insulin (μIU/mL) × fasting glucose (mmol/L)]/22.5. Plasma malondialdehyde (MDA) was measured using the TBARS assay kit (ZeptoMetrix Co., Buffalo, NY, USA). Urinary 8-epi-PGF_2α_ was measured via an enzyme immunoassay with a Urinary Isoprostane ELISA kit (Oxford Biomedical Research Inc., Rochester Hills, MI, USA). Finally, ba-PWV was measured with an automatic waveform analyzer (model VP-1000; Nippon Colin Ltd., Komaki, Japan).

### Affymetrix Axiom™ KORV1.0-96 Array hybridization

A total of 516 samples were genotyped using an Axiom® 2.0 Reagent Kit (Affymetrix Axiom® 2.0 Assay User Guide; Affymetrix, Santa Clara, CA, USA) according to the manufacturer’s protocol. Detailed procedures have been described previously^20^. Genotype data were produced using the Korean Chip (K-CHIP) available through the K-CHIP consortium. The K-CHIP was designed by the Center for Genome Science at the Korea National Institute of Health (4845-301, 3000–3031).

### Statistical analysis

Descriptive statistical analyses were performed using SPSS version 23.0 (IBM, Chicago, IL, USA). Skewed variables were transformed into their logarithmic forms for the statistical analysis. An independent *t*-test and a paired *t*-test were performed for continuous variables to compare parameters between the two groups and within groups. Frequencies were tested using a Chi-square test. A one-way ANOVA followed by the Bonferroni *post hoc* test were performed to compare differences among the *ESRRG* rs1980522 genotype groups in the NFG control and IFG groups. The general linear model UNIANOVA statistical procedure was applied to adjust the variables. Hardy-Weinberg equilibrium (HWE) was assessed using PLINK version 1.07 (http://pngu.mgh.harvard.edu/purcell/plink/). Associations between IFG and genotypes were calculated using the odds ratio (OR) [95% confidence intervals (CIs)] of a logistic regression model with adjustment for confounding factors. The mean values are expressed as the means ± standard error (SE). A two-tailed *P*-value < 0.05 was considered to indicate statistical significance.

## Results

The clinical and biochemical characteristics of the NFG controls (*n* = 374) and IFG cases (*n* = 142) at baseline are shown in Table 1. After adjusting for age, BMI, gender distribution, smoking and drinking, the IFG subjects had significantly higher systolic and diastolic BP, serum triglycerides, fasting glucose, HOMA-IR indices, plasma MDA, urinary 8-epi-PGF_2α_, and ba-PWV levels than the NFG subjects (Table 1). Over the 3.5-year period, the estimated macronutrient intake, total energy intake, and total energy expenditure did not change significantly in the NFG controls. The replacement of refined rice with whole grains was observed only in the IFG group (data not shown).

**Table 1:** Clinical and biochemical characteristics of the NFG controls and patients with IFG.

	Controls (*n* = 374)	IFG (*n* = 142)	*P*	*P’*
Age (years)	45.0 ± 0.47	48.7 ± 0.71	<0.001	—
BMI (kg/m^2^)	23.2 ± 0.14	24.1 ± 0.24	0.001	—
Male/female *n* (%)	188 (50.3)/186 (49.7)	65 (45.8)/77 (54.2)	0.362	—
Current smoker *n* (%)	85 (22.7)	30 (21.1)	0.696	—
Current drinker *n* (%)	241 (64.6)	96 (67.6)	0.523	—
Systolic BP (mmHg)	116.2 ± 0.69	121.2 ± 1.02	<0.001	0.005
Diastolic BP (mmHg)	71.2 ± 0.53	75.5 ± 0.81	<0.001	0.001
Triglycerides (mg/dL)^*∮*^	94.9 ± 2.67	126.8 ± 5.64	<0.001	<0.001
Total cholesterol (mg/dL)^*∮*^	187.6 ± 1.64	196.3 ± 2.75	0.007	0.278
HDL cholesterol (mg/dL)^*∮*^	53.7 ± 0.73	51.7 ± 1.18	0.123	0.180
LDL cholesterol (mg/dL)^*∮*^	114.9 ± 1.56	119.2 ± 2.55	0.181	0.942
Glucose (mg/dL)^*∮*^	89.3 ± 0.33	106.0 ± 0.46	<0.001	<0.001
Insulin (μIU/dL)^*∮*^	8.49 ± 0.16	9.49 ± 0.60	0.234	0.835
HOMA-IR^*∮*^	1.88 ± 0.04	2.51 ± 0.17	<0.001	<0.001
MDA (nmol/mL)^*∮*^	8.98 ± 0.13	10.2 ± 0.30	0.004	0.001
8-epi-PGF_2α_ (pg/mg creatinine)^*∮*^	1380.7 ± 31.0	1495.9 ± 46.1	0.003	0.017
ba-PWV (cm/s)^*∮*^	1271.9 ± 8.92	1347.9 ± 15.5	<0.001	0.017

Means ± SE. ^*∮*^Tested by logarithmic transformation. An independent *t*-test was used to calculate the *P*-values. The *P’*-values were calculated after adjusting for age, BMI, gender, smoking, and drinking.

### Distribution of the *ESRRG* rs1890552 A > G polymorphism

The observed and expected frequencies of the *ESRRG* rs1890552 A > G polymorphism were in HWE in the entire population and in the NFG and IFG groups. A significant difference in the distribution of *ESRRG* rs1890552 A > G genotypes was observed between the NFG and IFG groups (*P* = 0.039). The *ESRRG* rs1890552 A > G genotypes consisted of 29.9% AA, 50.3% AG and 25.8% GG in the NFG controls and 26.8% AA, 43.0% AG and 30.3% GG in the IFG group. The G allele frequency differed significantly between the NFG controls (44.9%) and the IFG group (51.8%) (*P* = 0.029).

The presence of the GG genotype of the *ESRRG* rs1890552 A > G SNP was associated with a higher risk of IFG before [OR: 1.761 (95% CI: 1.1135–2.732), *P* = 0.012] and after adjustment for age, BMI, smoking, drinking, and systolic and diastolic BP [OR: 1.810 (95% CI: 1.141–2.872), *P* = 0.012]. Similarly, the presence of the G allele of the *ESRRG* rs1890552 A > G SNP was associated with a higher risk of IFG before [OR: 1.316 (95% CI: 1.001–1.730), *P* = 0.049] and after adjustment for age, BMI, smoking, drinking, and systolic and diastolic BP [OR: 1.360 (95% CI: 1.022–1.810), *P* = 0.035]. The ORs of G allele were somewhat lower than the GG genotype of the *ESRRG* rs1890552 A > G SNP.

### Influence of the *ESRRG* rs1890552 A > G polymorphism on fasting glucose, ba-PWV, and urinary 8-epi-PGF_2α_ before and after the 3.5-year follow-up

**Figure 1:**
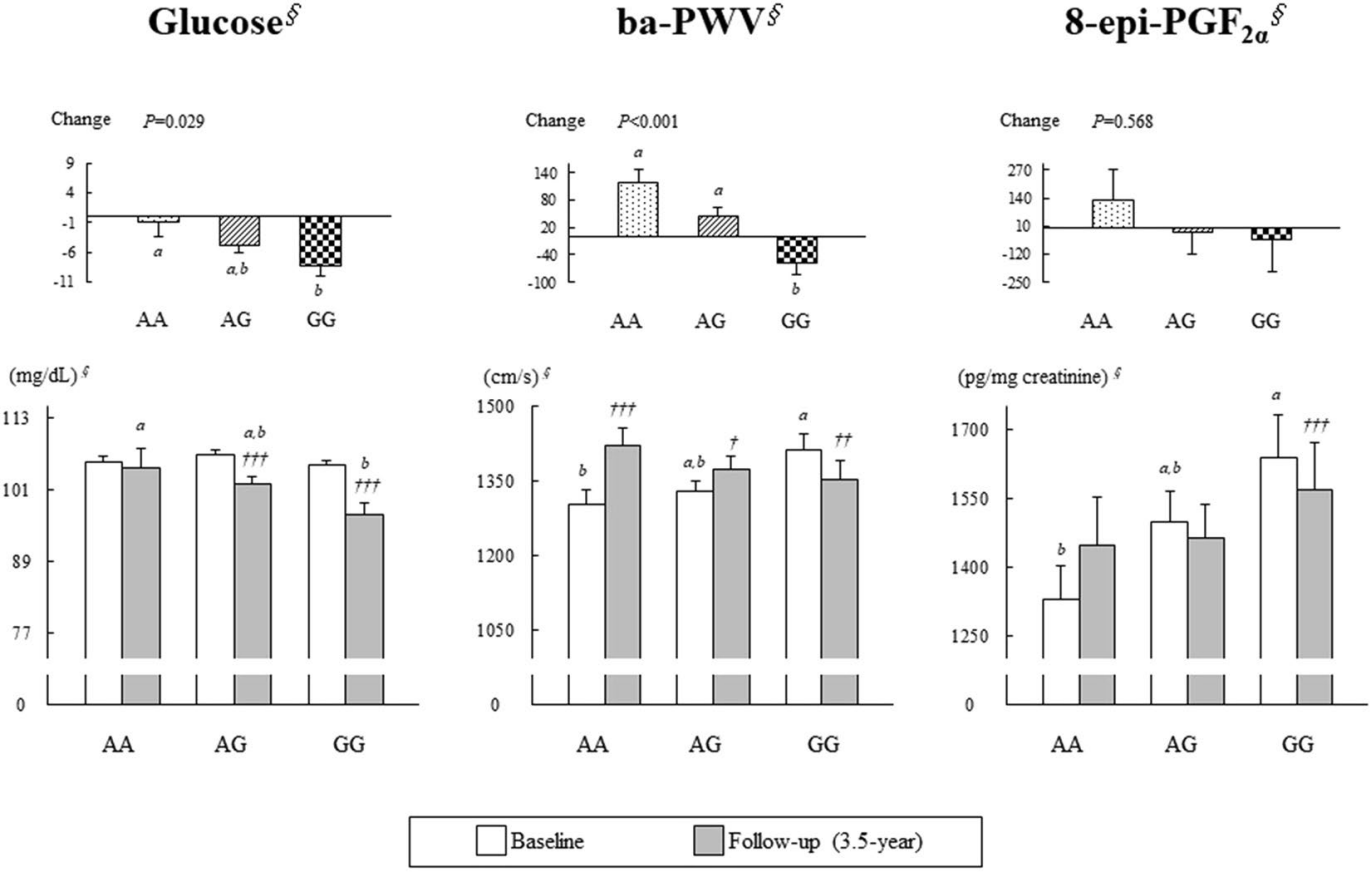
Influence of *ESRRG* rs189055 A > G genotype on fasting glucose, ba-PWV, and 8-epi-PGF_2α_ before and after a 3.5-year follow-up in subjects with IFG. Means ± SE. ^*∮*^Tested by logarithmic transformation. One-way ANOVA was used to calculate the *P*-values. All letters indicating *P* < 0.05 were derived from the Bonferroni *post hoc* test. Comparisons without a significant difference are indicated with the same letter, and significant differences are indicated with a different letter. Changes in *P*-values were adjusted for the baseline value. ^†^ *P* < 0.05, ^††^ *P* < 0.01, and ^†††^ *P* < 0.001 derived from a paired *t*-test in each genotype.

Table 2 and Fig. 1 show the influence of the *ESRRG* rs1890552 A > G genotypes on fasting glucose, ba-PWV, and urinary 8-epi-PGF_2α_ before and after the 3.5-year follow-up. At the 3.5-year follow-up, the NFG controls with the AG genotype showed a significant increase in fasting glucose from baseline (Table 2). In the IFG group, the AG and GG subjects showed a significant reduction in fasting glucose (Fig. 1). This glucose reduction was greater in the GG subjects than in the AG subjects. A significant association was observed between changes (compared to baseline) in fasting glucose and *ESRRG* rs1890552 A > G genotype in the IFG subjects before and after adjusting for the baseline values (AA: −0.83 ± 2.61 mg/dL, AG: −4.85 ± 1.31 mg/dL, and GG: −8.33 ± 1.74 mg/dL; *P* = 0.029) (Fig. 1).

**Table 2:** Association of *ESRRG* rs1890552 genotypes with glucose, ba-PWV, and 8-epi-PGF_2α_ in the NFG controls and patients with IFG.

	**Controls (** ***n*** **=374)**			***P***	***P’***	**IFG (** ***n*** = **142)**			***P***	***P’***
	**AA (** ***n*** = **112)**	**AG (** ***n*** = **188)**	**GG (** ***n*** = **74)**			**AA (** ***n*** = **38)**	**AG (** ***n*** = **61)**	**GG (** ***n*** = **43)**		
Age (years)	44.6 ± 0.85	45.1 ± 0.67	45.6 ± 1.04	0.776		50.8 ± 1.44	47.9 ± 1.04	48.1 ± 1.27	0.195	
BMI (kg/m^2^)	23.2 ± 0.26	23.1 ± 0.21	23.3 ± 0.28	0.864		24.0 ± 0.53	24.4 ± 0.31	23.8 ± 0.50	0.509	
Change in weight (kg)	0.59. ± 0.25	0.63 ± 0.23	0.61 ± 0.35	0.992	0.990	−0.15 ± 0.46	0.35 ± 0.38	0.11 ± 0.28	0.660	0.558
Glucose (mg/dL)^∮^										
Before	89.3 ± 0.54	88.9 ± 0.48	90.4 ± 0.75	0.232		105.6 ± 0.98	106.8 ± 0.73	105.2 ± 0.67	0.332	
After	90.2 ± 0.76	91.1 ± 0.68^†^	90.2 ± 0.92	0.746		104.8 ± 3.00^a^	101.9 ± 1.43^a,b,†††^	96.9 ± 1.94^b,†††^	0.022	
Change	0.90 ± 0.68	2.21 ± 0.71	−0.19 ± 0.99	0.115	0.286	−0.83 ± 2.61^a^	−4.85 ± 1.31^a,b^	−8.33 ± 1.74^b^	0.027	0.029
ba-PWV (cm/s)^∮^										
Before	1261.4 ± 16.8	1278.7 ± 12.5	1270.7 ± 19.7	0.652		1304.1 ± 28.5^b^	1328.8 ± 20.6^a,b^	1413.7 ± 31.8^a^	0.020	
After	1283.3 ± 19.5	1297.9 ± 13.9	1296.0 ± 20.0	0.732		1422.6 ± 32.9^†††^	1374.3 ± 25.8^†^	1354.3 ± 38.9^††^	0.263	
Change	21.9 ± 11.1	19.2 ± 8.76	25.3 ± 14.2	0.931	0.950	118.5 ± 28.7^a^	45.5 ± 17.0^a^	−59.4 ± 23.9^b^	<0.001	<0.001
8-epi-PGF_2α_ (pg/mg creatinine)^∮^										
Before	1238.6 ± 49.4^b^	1427.9 ± 47.1^a^	1472.5 ± 64.0^a^	0.007		1328.7 ± 75.2^b^	1498.7 ± 67.8^a,b^	1639.2 ± 93.6^a^	0.020	
After	1374.4 ± 53.4^†^	1362.9 ± 42.4	1413.4 ± 57.3	0.569		1446.5 ± 105.5	1462.9 ± 73.5	1570.2 ± 102.3	0.666	
Change	149.8 ± 70.8	−47.4 ± 59.5	−58.8 ± 83.2	0.079	0.841	129.2 ± 140.2	−17.2 ± 104.5	−49.1 ± 148.9	0.630	0.568

Means ± SE. ^*∮*^Tested by logarithmic transformation. One-way ANOVA was used to calculate the *P*-values. The *P’*-values were adjusted for baseline values. All letters representing *P*-values < 0.05 were derived from the Bonferroni *post hoc* test; no significant differences are present for comparisons marked with the same letter, and significant differences are indicated with a different letter. ^†^ *P* < 0.05, ^††^ *P* < 0.01, and ^†††^ *P* ^*< *^0.001 derived from paired *t*-tests before and after the follow-up period in each genotype.

At baseline, a significant association was observed between ba-PWV and *ESRRG* rs1890552 A > G genotype in the IFG group (AA: 1304 ± 29 cm/s, AG: 1329 ± 21 cm/s, and GG: 1414 ± 32 cm/s; *P* = 0.020) (Fig. 1). In the IFG group, the GG subjects showed a significantly higher ba-PWV than the AA subjects at baseline. At the 3.5-year follow-up, subjects with either the AA or AG genotype showed a significant increase in ba-PWV, whereas the GG subjects showed a significant decrease compared to the baseline. A significant association was observed between changes in ba-PWV and *ESRRG* rs1890552 A > G genotype in the IFG group (AA: 119 ± 29 cm/s, AG: 46 ± 17 cm/s, and GG: −59 ± 24 cm/s; *P* < 0.001). A significant difference in changes in ba-PWV was found between the IFG subjects with AA and GG, as well as between those with AG and GG. At the 3.5-year follow-up, there were no significant differences in ba-PWV among the subjects with IFG according to their *ESRRG* rs1890552 A > G genotype (Fig. 1).

At baseline, a significant association was observed between urinary 8-epi-PGF_2α_ and *ESRRG* rs1890552 A > G genotype in the NFG control and IFG groups (Table 2), with the GG subjects showing significantly higher urinary 8-epi-PGF_2α_ than the AA subjects.

### Association between the *ESRRG* rs1890552 A > G polymorphism and dietary advice-mediated changes per allele

**Figure 2:**
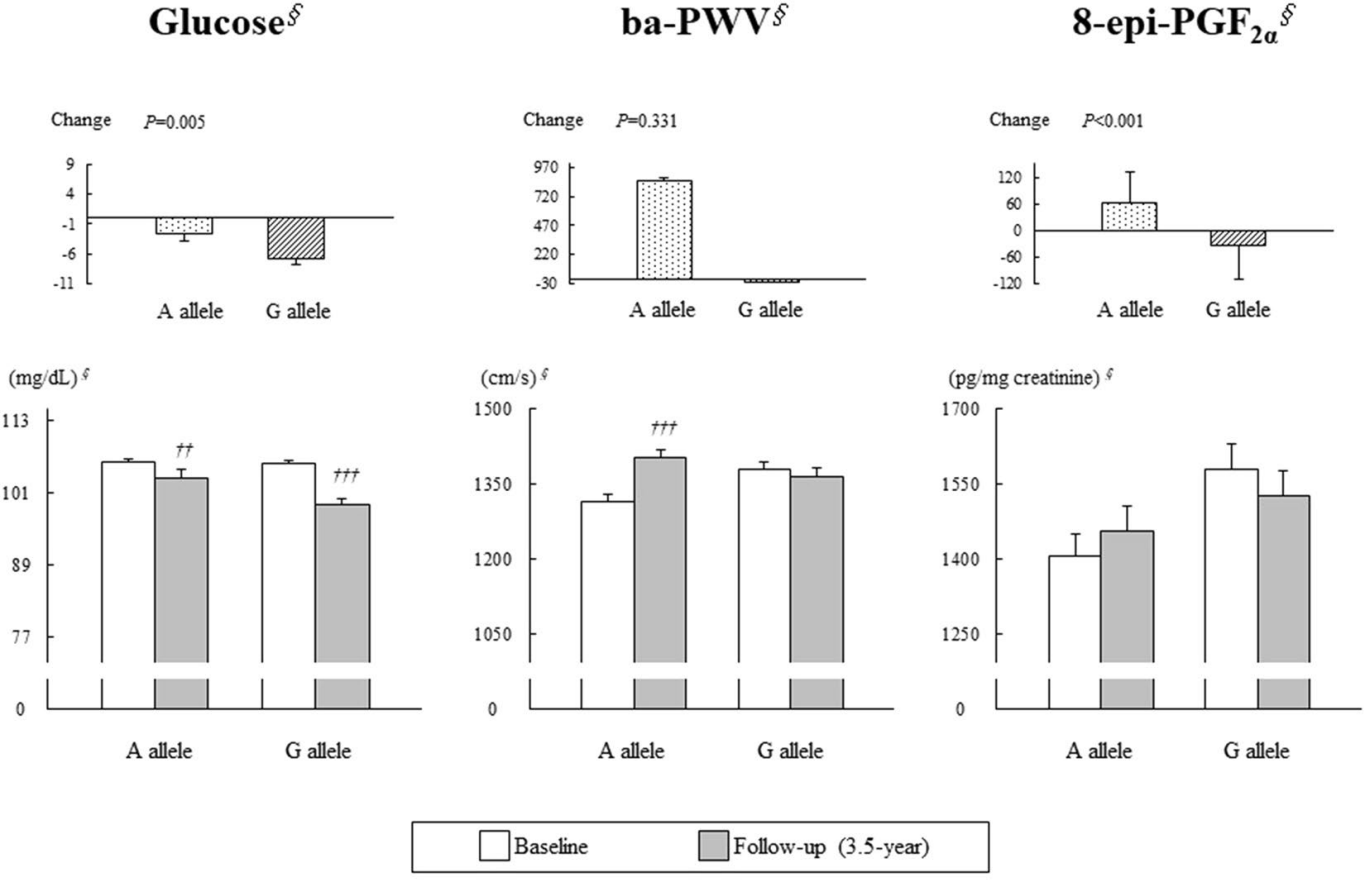
Influence of *ESRRG* rs189055 A > G alleles on fasting glucose, ba-PWV, and 8-epi-PGF_2α_ before and after a 3.5-year follow-up in subjects with IFG. Means ± SE. ^*∮*^Tested by logarithmic transformation. An independent *t*-test was used to calculate the *P*-values. Changes in *P*-values were adjusted for the baseline value. ^†^ *P* < 0.05, ^††^ *P* < 0.01, and ^†††^ *P* < 0.001 derived from a paired *t*-test in each allele.

Table 3 and Fig. 2 show the influence of the *ESRRG* rs1890552 A > G alleles on fasting glucose, ba-PWV, and urinary 8-epi-PGF_2α_ before and after the 3.5-year follow-up. After the 3.5-year follow-up, the fasting glucose of all the subjects in the IFG group was significantly decreased; however, this reduction was more pronounced in subjects with a G allele (Fig. 2). Moreover, the changes in fasting glucose were significantly different between subjects in the IFG group with an A allele and those with a G allele both before and after adjusting for baseline values. The baseline levels of ba-PWV in subjects with a G allele were significantly elevated in both the NFG control and IFG groups. After the subjects in the IFG group received dietary advice, those with a G allele showed a slight reduction in ba-PWV (−15.9 ± 12.8 cm/s) (Table 3), whereas those with the GG genotype showed a significantly greater reduction (−59.4 ± 23.9 cm/s, *P* = 0.007) (Table 2). In the IFG group, a significant difference in the changes in 8-epi-PGF_2α_ was observed between the subjects with an A allele and those with a G allele (61.4 ± 71.6 vs. −35.4 ± 74.4 pg/mg creatinine) both before and after adjusting for baseline values (Fig. 2).

**Table 3:** Association of *ESRRG* rs1890552 alleles with glucose, ba-PWV, and 8-epi-PGF_2α_ in the NFG controls and subjects with IFG.

	**Controls (** ***n*** = **374)**		***P***	***P’***	**IFG (** ***n*** = **155)**		***P***	***P’***
	**A allele**	**G allele**			**A allele**	**G allele**		
Age (years)	44.8 ± 0.45	45.3 ± 0.50	0.481		49.5 ± 0.73	48.0 ± 0.67	0.125	
BMI (kg/m^2^)	23.2 ± 0.14	23.2 ± 0.14	0.951		24.2 ± 0.25	24.0 ± 0.24	0.701	
Change in weight (kg)	0.61 ± 0.14	0.62 ± 0.17	0.951	0.908	0.07 ± 0.25	0.21 ± 0.19	0.670	0.796
Glucose (mg/dL)^∮^								
Before	89.1 ± 0.30	89.6 ± 0.36	0.381		106.1 ± 0.50	105.9 ± 0.41	0.718	
After	90.6 ± 0.43^††^	90.7 ± 0.48	0.886		103.5 ± 1.33^††^	99.0 ± 1.01^†††^	0.004	
Change	1.50 ± 0.42	1.15 ± 0.51	0.594	0.862	−2.62 ± 1.18	−6.88 ± 0.91	0.004	0.005
ba-PWV (cm/s)^∮^								
Before	1269.3 ± 8.60	1275.2 ± 9.26	0.003		1315.1 ± 14.4	1378.5 ± 16.0	0.003	
After	1290.0 ± 9.81	1297.0 ± 9.94	0.528		1401.1 ± 17.3^†††^	1362.6 ± 19.2	0.387	
Change	20.7 ± 5.84	21.9 ± 6.58	0.056	0.884	860 ± 13.8	−15.9 ± 12.8	0.351	0.311
8-epi-PGF_2α_ (pg/mg creatinine)^∮^								
Before	1325.6 ± 29.1	1447.6 ± 33.0	0.393		1405.5 ± 42.6	1580.1 ± 47.8	0.083	
After	1369.0 ± 28.1^†^	1384.5 ± 29.8	0.429		1454.0 ± 52.2	1524.9 ± 51.8	0.135	
Change	57.3 ± 38.8	−52.3 ± 42.2	0.892	0.825	61.4 ± 71.6	−35.4 ± 74.4	<0.001	<0.001

Means ± SE. ^∮^Tested by logarithmic transformation. An independent *t*-test was used to calculate the *P*-values. The *P’*-values were adjusted for baseline values. ^†^ *P* < 0.05, ^††^ *P* < 0.01, and ^†††^ *P* < 0.001 derived from paired *t*-tests before and after the follow-up period in each allele.

## Discussion

The most relevant finding from the present study is that the *ESRRG* rs1890552 A > G polymorphism influences the ability of dietary advice to alter arterial stiffness, as measured by ba-PWV, in subjects with IFG. Additionally, in the IFG group, the GG subjects showed a significant decrease in fasting glucose after the 3.5-year follow-up, which was greater than the reduction in the AG subjects. Moreover, subjects in the IFG group with a G allele presented a more pronounced glucose reduction. These results suggest a role of the *ESRRG* rs1890552 A > G polymorphism in modulating interindividual differences in arterial stiffness, with a reduction in fasting glucose in response to dietary advice in subjects with IFG after 3.5 years of follow-up. The PWV is an established index of arterial stiffness^22^, and the ba-PWV has characteristics similar to the central aortic PWV^23^. The underlying mechanisms responsible for arterial stiffness are unknown; however, slightly high-normal glucose levels were reported to be associated with arterial stiffness measured using ba-PWV^24^. Indeed, IFG is known to be a risk factor for arterial stiffness and CVD, and the ba-PWV value in the IFG group was significantly higher than the value in the NFG group^24^. This study also showed a higher ba-PWV in subjects with IFG than in the NFG subjects. Additionally, the G allele, particularly the GG genotype, which had a higher risk of IFG than the AA and AG genotypes, showed a significantly higher ba-PWV than an A allele at baseline.

Arterial stiffness, which is one of the most significant manifestations of vascular aging^25, 26^, can increase with age, even in healthy individuals without clinical CVD^27^. At the 3.5-year follow-up, IFG subjects with either AA or AG showed significant increases in the ba-PWV, whereas the subjects with GG showed a significant decrease from baseline. Thus, at the 3.5-year follow-up, significant differences in ba-PWV disappeared among the subjects with IFG according to their *ESRRG* rs1890552 A > G genotype. On the other hand, only subjects with an A allele in the IFG group showed a significant increase in ba-PWV. At the 3.5-year follow-up, the ba-PWV of subjects with a G allele decreased, but this decrease was not statistically significant. Based on these results, the effect of dietary advice was more profound in individuals homozygous for the minor allele (the GG genotype) than in subjects with a G allele. The significant decrease in ba-PWV in the GG subjects may be partially due to the greater reduction in fasting glucose than in the AA genotype. The drop in fasting glucose was less pronounced among subjects with a G allele than in those with the GG genotype; therefore, the impact of this SNP on ba-PWV was not that statistically significant, although genotypic variation was observed. Indeed, a positive correlation was observed between changes in fasting glucose and changes in ba-PWV in the subjects with IFG (*r* = 0.385, *P* < 0.001).

The most available and reliable marker of oxidative stress *in vivo* has been revealed to be 8-epi-PGF_2α_^28, 29^, which is one of the stable products produced from the non-cyclooxygenase peroxidation of arachidonic acid and has been suggested to be a highly precise and reliable predictor of glycemic control and oxidative status in patients with T2D^18, 30^ and IGT^19^. A clear positive correlation has been demonstrated among oxidative stress, IR, and prediabetes in humans^31^. In the present study, a significant association was observed between urinary 8-epi-PGF_2α_ and the *ESRRG* rs1890552 A > G polymorphism in NFG and IFG subjects at baseline. The GG subjects showed a significantly higher urinary 8-epi-PGF_2α_ than the AA subjects in both the NFG and IFG groups at baseline, which was in line with our previous findings^20^. The subjects in the IFG group with a G allele showed significantly different changes in 8-epi-PGF_2α_; however, differences based on genotype were not observed. Although there was no significant association between changes in 8-epi-PGF_2α_ and *ESRRG* rs1890552 A > G genotype among subjects in the IFG group, the direction of the change in 8-epi-PGF_2α_ among individuals who received dietary advice was the same as for ba-PWV.

Our study design has some limitations that should be discussed. First, dietary intake was based on self-reports obtained from weighed food. However, measurement errors from self-reported dietary intake and lifestyle variables have been shown to be relatively small^32^. In this study, the well-controlled fasting glucose concentration in the IFG group reflected the compliance of the subjects with the dietary intervention. Second, due to the small sample size, the genetic analysis results should be interpreted with caution; therefore, further additional studies with a larger sample are needed to confirm the present findings. Despite these limitations, this study showed that the *ESRRG* rs1890552 A > G polymorphism could affect changes in arterial stiffness in response to dietary advice in a 3.5-year prospective study in patients with IFG. Subjects with the *ESRRG* rs1890552 G allele, specifically the GG subjects in the IFG group who showed a decrease in fasting glucose at the 3.5-year follow-up, exhibited greater reductions in ba-PWV.

## Conclusions

This study suggests that the *ESRRG* rs1890552 A > G polymorphism can modulate interindividual differences in atrial stiffness, with a reduction in fasting glucose in response to dietary advice in subjects with IFG after 3.5 years of follow-up. Based on our in-depth analysis of genetic differences in fasting glucose, ba-PWV, and 8-epi-PGF_2α_, the size of the effects differed based on the *ESRRG* rs1890552 A > G polymorphism. These results provide good evidence for the tailoring of dietary advice to individuals based on their genetic patterns.
